# Patterns of remission from alcohol dependence in the United Kingdom: results from an online panel general population survey

**DOI:** 10.1186/s13011-023-00588-1

**Published:** 2024-01-04

**Authors:** John A. Cunningham, Christina Schell, Hollie Walker, Alexandra Godinho

**Affiliations:** 1 National Addiction Centre, Institute of Psychiatry, Psychology and Neuroscience, Kings College London, London, UK; 2 Centre for Addiction and Mental Health, Toronto, Canada; 3 Department of Psychiatry, University of Toronto, Toronto, Canada; 4 Humber River Health Research Institute, Humber River Health, Toronto, Canada

**Keywords:** Alcohol, Epidemiology, Treatment, Abstinence, Moderate drinking, Remission, Recovery

## Abstract

**Background:**

Previous research has demonstrated that remissions from alcohol use disorders can occur without accessing treatment. The current study explored the prevalence of such untreated remissions in the UK and further, examined the extent to which people who resolved an alcohol use disorder regarded themselves as ever, or currently, being in recovery.

**Methods:**

Participants were recruited using the Prolific online platform. Participants who met criteria for lifetime alcohol dependence (ICD-10) were asked about their drinking at its heaviest, use of treatment services, whether they identified as being in recovery, and their current alcohol consumption (to identify those who were abstinent or drinking in a moderate fashion).

**Results:**

A total of 3,994 participants completed surveys to identify 166 participants with lifetime alcohol dependence who were currently abstinent (n = 67) or drinking in a moderate fashion (n = 99). Participants who were currently abstinent were more likely to have accessed treatment than those who were currently moderate drinkers (44.4% versus 16.0%; Fischer’s exact test = 0.001). Further, those who were abstinent were heavier drinkers prior to remission [Mean (SD) drinks per week = 53.6 (31.7) versus 29.1 (21.7); t-test = 5.6, 118.7 df, *p* < .001] and were more likely to have ever identified themselves as ‘in recovery’ (51.5% versus 18.9%; Fischer’s exact test = 0.001) than current moderate drinkers.

**Conclusions:**

While participants with an abstinent remission were more likely than those currently drinking in a moderate fashion to have accessed treatment and to identify as being ‘in recovery,’ the majority of participants reduced their drinking without treatment (and did not regard themselves as in recovery).

**Supplementary Information:**

The online version contains supplementary material available at 10.1186/s13011-023-00588-1.

## Background

The common view of remission from alcohol dependence is that treatment (including attendance at mutual aid, i.e. Alcoholics Anonymous; AA) is required and that abstinence is the only successful outcome [1]. However, there is evidence that there are multiple pathways to remission, including addressing alcohol concerns without treatment and drinking in a moderate fashion. This literature includes epidemiological studies reporting on the prevalence of different pathways to remission in the general population (conducted in the USA, Canada, Germany, and Sweden) [2–7]. Other studies have recruited samples of former heavy drinkers and incorporated in depth interviews to identify factors associated with change [8, 9].

The current study contributes to this literature in two ways. First, while there are findings from the UK reporting on the small proportion of people with alcohol concerns who ever accessed treatment [10], there appears to be no existing study reporting on prevalence of untreated and moderate drinking remissions using UK data. Having results from multiple countries, including the UK, on the same topic can increase support for the existence of untreated and moderate drinking remissions. Second, the current study provides new information on the proportion of people who ever regarded themselves as being in recovery from alcohol problems. Our prior work on this topic indicated that former heavy drinkers in the UK might be less likely to regard themselves as ever, or currently, being in recovery compared to former heavy drinkers in the USA [11]. However, this work was limited due to the use of a sample that was not representative of the general population. Given the predominance of the twelve-step recovery narrative in discussions regarding the best ways to provide addictions treatment [12–15], it is important to establish the proportion of former heavy drinkers who might not identify with this approach to treatment provision. This is because treatments that match alcohol consumers’ perspectives on the nature of their problem may prove more attractive to those hesitating about seeking care [11].

The aims of the study are: (1) to explore the patterns of treated and untreated remission from lifetime alcohol dependence in a representative sample from the United Kingdom; and (2) to assess the proportion of these participants who currently or ever regarded themselves as being in recovery.

## Methods

Participants were recruited from the Prolific online website using the option to recruit a sample whose distribution mirrors the age, sex, and ethnicity of the general population of the UK who were 18 years or older [16]. Prospective participants saw an advertisement asking them to complete a ‘short survey about drinking alcohol.’ They were told that the survey would take approximately 20–25 min to complete, that they did not need to be a current or past drinker to participate and that they would be paid £4 into their Prolific user account. After reading an information sheet and agreeing to take part in the study, participants completed a series of questions to identify the subsample who were currently either abstinent or drinking in a moderate fashion (definition provided below), and who used to drink more heavily.

Past heavy drinking was defined broadly with participants being asked if they had ever consumed six or more drinks per occasion at least once per week for a month or more [7, 17]. Further, to identify those participants with substantial alcohol concerns prior to reducing their drinking, only those who met criteria for lifetime alcohol dependence (ICD-10) were included in the present analyses. The scale employed to assess lifetime alcohol dependence was originally developed for use in a general population survey (the Ontario Drug Monitor) [18]. The scale asks if the participants experienced each of the criteria used to assess ICD-10 alcohol dependence (please see Supplementary Material 1 for a copy of the entire survey used in the current study) and the measure of lifetime alcohol dependence reflects occurrence of symptoms over the person’s lifetime rather than experience of symptoms all within the same year. Participants were defined as being in remission from lifetime alcohol dependence if they reported being abstinent in the past year or reported drinking a in a moderate fashion. Using criteria developed in our previous research [5, 7, 19], current moderate drinking was defined as usually drinking two or less drinks per drinking occasion (drink defined as one UK unit); drinking six or more drinks on one occasion, less than once per month; and never drinking more than eight drinks on one occasion in the past year. A figure outlining how many drinks (i.e., units) were in different types of beverages was included to promote more accurate reporting of alcohol consumption.

Participants were asked a series of questions that included amount of alcohol consumption during their heaviest period (please see Table 1 for items assessed) and prior use of treatment. Treatment use was assessed in two fashions: (1) by asking participants if they had, ‘ever gone to Alcoholics Anonymous, or any other community agency or seen a physician, counselor, or any other professional for a reason that was related in any way to your drinking?’ [2, 3]; and (2) asking if they had ever, ‘had contact with, or used any of the following, specifically for alcohol concerns?’ Ever treatment use was defined as answering yes to the global treatment use question or by stating that they had ever talked to someone at their GP surgery (e.g., doctor or nurse), or to a community pharmacist about their alcohol consumption. The items about treatment use contained an attention check question, ‘I want to indicate that I have read this question by checking never’ [20, 21].

In order to explore views on being ‘in recovery’, participants were asked a series of three questions taken from the Kelly et al. [22] general population survey on recovery (modified to ask just about alcohol): (a) Did you used to have a problem with alcohol but no longer do?; (b) Do you consider yourself to be in recovery?; and (c) Did you ever consider yourself to be in recovery? The survey ended with a series of demographic questions and a final item asking if they answered all questions truthfully (1 = strongly disagree; 7 = strongly agree).

### Post-stratification weighting

Initial examination of age distribution of the sample recruited indicated that it was skewed to younger participants. As such, a post-stratification weighting was applied, using data from the Office of National Statistics estimate of the population for the United Kingdom in July of 2021 [23, 24]. Participants who did not identify as male or female were assigned a weight of 1 in order to retain them in the sample. Percentages, means and standard deviations are reported based on weighted data. Sample sizes are reported as unweighted data. Inferential analyses were also repeated without post-stratification and it was observed that there were no variations in the results of the analyses that would lead to a different interpretation of the results than the ones described based on the use of the stratified data (analyses not sown here).

### Ethics approval

The study received ethics approval from the REB of King’s College London. As this was an anonymous online panel survey, participants provided consent to participate by checking that they agreed to complete the study after reading an information sheet describing the research.

## Results

A total of 3,994 participants completed the survey and provided their Prolific ID. Of these participants, 3,749 answered the attention check questions correctly and affirmed that they strongly agreed that they had answered all questions truthfully. Of these, 802 participants met criteria for lifetime alcohol dependence and, of these, 166 were currently abstinent or drinking in a moderate fashion. Table 1 displays the demographic characteristics of these 166 participants, separated by current drinking status (abstinent n = 67, or moderate drinker n = 99). Participants who were currently abstinent were older [Mean (SD) Age = 58.5 (12.4) versus 51.5 (13.4); t-test = 3.4, 164 df, *p* < .001; Cohen’s *d* = 0.53], less likely to be married or in a common law relationship (50.0% versus 71.3%; Fischer’s exact test = 0.006; Cramer’s *V* = 0.22), and more likely to have a family income of less than £30,000 (59.7% versus 34.4%; Fischer’s exact test = 0.002; Cramer’s *V* = 0.25) compared to those who reduced their drinking to a moderate level.

**Table 1:** Current demographic characteristics of former heavy drinkers who met criteria for lifetime alcohol dependence

	Abstinent	Moderate	*p*
	(n = 67)	(n = 99)	
Mean (SD) Age	58.5 (12.4)	51.5 (13.4)	0.001
% Male	52.8	54.3	0.876
% Married/Common Law	50.0	71.3	0.006
% Completed education after turning 19	59.7	59.6	1.00
% Full/part time employed	48.6	61.7	0.115
% Family income < £30,000	59.7	34.4	0.002

Table 2 displays the drinking of participants during their heaviest period and the experience of ICD-10 symptoms. Participants who were abstinent drank more during their heaviest period [Mean (SD) drinks per week = 53.6 (31.7) versus 29.1 (21.7); t-test = 5.6, 118.7 df, *p* < .001; Cohen’s *d* = 0.92], reported a larger amount for their heaviest drinking occasion [Mean (SD) = 18.4 (11.0) versus 12.9 (5.9); t-test = 3.8, 101.9 df, *p* < .001; Cohen’s *d* = 0.64], and were more likely to report consuming 12 or more drinks on one occasion at least once per week (63.4% versus 35.9%; Fischer’s exact test = 0.001; Cramer’s *V* = 0.27) compared to those who reduced their drinking to a moderate level. In addition, current abstinent participants versus moderate drinking participants reported experiencing a larger number of ICD-10 dependence symptoms [Mean (SD) = 6.8 (2.5) versus 4.7 (1.9); t-test = 6.04, 128.1 df, *p* < .001; Cohen’s *d* = 0.98]. The two groups did not differ significantly in the recency of their last symptom (*p* = .393).

**Table 2:** Drinking from heaviest period of alcohol consumption

	Abstinent	Moderate	*p*
	(n = 67)	(n = 99)	
Mean (SD) drinks per week	53.6 (31.7)	29.1 (21.7)	0.001
Mean (SD) largest number drinks	18.4 (11.0)	12.9 (5.9)	0.001
% 12 + drinks at least once per week	63.4	35.9	0.001
Mean (SD) number of symptoms (ICD-10)	6.8 (2.5)	4.7 (1.9)	0.001
Mean (SD) recency of symptoms (years)	12.9 (12.8)	13.5 (15.4)	0.393

Table 3 provides a summary of the participants’ characteristics of change. Participants who were currently abstinent were more likely to endorse that they had a problem with their drinking and now no longer do (86.8% versus 46.8%; Fischer’s exact test = 0.001; Cramer’s *V* = 0.42) and to rate that they had a major or very major problem when their drinking was at its heaviest (68.1% versus 28.4%; Fischer’s exact test = 0.001; Cramer’s *V* = 0.39), compared to those who were currently moderate drinkers. Further, those who were currently abstinent were more likely to identify as currently being in recovery (44.1% versus 10.8%; Fischer’s exact test = 0.001; Cramer’s *V* = 0.38) and to endorse that they had ever been in recovery (51.5% versus 18.9%; Fischer’s exact test = 0.001; Cramer’s *V* = 0.34). Finally, those who were currently abstinent were more likely to endorse that they had ever accessed alcohol treatment compared to those who were current moderate drinkers (44.4% versus 16.0%; Fischer’s exact test = 0.001; Cramer’s *V* = 0.31).

**Table 3:** Characteristics of change

	Abstinent	Moderate	*p*
	(n = 67)	(n = 99)	
% Had problem, now no longer do	86.8	46.8	0.001
% Major/very major problem when drinking at heaviest	68.1	28.4	0.001
% Identify as in recovery	44.1	10.8	0.001
% Identify as ever in recovery	51.5	18.9	0.001
% Alcohol treatment	44.4	16.0	0.001

## Discussion

The current project explored remission from alcohol dependence in the UK. Several patterns emerged that were similar to those found in other countries (USA, Canada, Germany, Sweden) [2–7]. Of particular note, the majority of participants stated that they had never accessed treatment, including Alcoholics Anonymous, or spoke to a GP or pharmacist about their alcohol consumption. While a larger proportion of those who reported not drinking in the past year endorsed some type of treatment use compared to those who had reduced to moderate drinking levels, less than half of participants with current abstinence reported ever using treatment. Also of note, a larger proportion of participants reported moderate drinking in the past year as opposed to abstinence. These patterns of change stand in contrast to established views about the steps people must take in order to recover from alcohol dependence, where abstinence is required and treatment is necessary [1].

Looking at the characteristics of participants, it appears that those who were currently abstinent had more severe alcohol use concerns prior to remission compared to those who were now moderate drinkers. While all participants met criteria for lifetime alcohol dependence, those who were currently abstinent reported experiencing more ICD-10 symptoms compared to those who were currently moderate drinkers. In addition, those who were currently abstinent reported larger amounts of alcohol consumption at the time of their heaviest use compared to those who were currently moderate drinkers. Combined with findings that current abstainers were more likely to have accessed treatment, this pattern of results makes conceptual sense with those who had more severe alcohol consumption being more likely to access treatment and to successfully address their alcohol concerns through abstinence rather than with moderate alcohol consumption [3, 25, 26]. Further research should examine whether factors, such as social capital, are related to these different patterns of remission [27]. In addition, consideration should be given on ways to measure other motivators for change that may have influenced participant’s remissions from alcohol concerns (e.g., family, friends, employer).

Another finding that contradicts current assumptions about how people resolve their alcohol concerns has to do with participants’ perceptions of whether they ever had a problem and if they currently, or had ever, regarded themselves as being ‘in recovery.’ Some research in this area has recruited only those people who stated they used to have a problem with alcohol when examining patterns of recovery [22, 28]. However, this approach assumes that, in order to be in recovery, one must first recognize having had a problem. Or, perhaps that everyone with severe alcohol concerns (e.g., such as those with alcohol dependence) must agree that they have a problem. The current analysis of a representative sample mirrors that of an earlier project using a convenience sample of people answering a survey about alcohol use, in that not everyone endorsed that they had an alcohol problem and now no longer do. As before, the large majority of participants who were abstinent regarded themselves as having had a problem and now no longer do. Less than half who reduced to moderate drinking levels regarded themselves as having had a problem (and now no longer do). This might reflect that their alcohol consumption was, on average, less severe than participants who were currently abstinent, or that they have been less exposed to 12-step ideas about recovery. An alternate explanation is that an association with addiction specific terminologies is stigmatising and contributes to reduced problem identification in general amongst lower intensity drinkers [29, 30]. One final explanation that cannot be discarded based on this data is that some participants do not endorse this question because they do not agree with the ‘no longer do’ part of the ‘had a problem (and now longer do)’ item.

Related to these findings, while participants who were currently abstinent were more likely than those who were currently moderate drinkers to view themselves as ever or currently being in recovery, the majority of participants did not apply the 12-step recovery concept to themselves. This may have significant implications for the types of treatment being offered to people who are concerned about their alcohol consumption. While some treatment modalities have a 12-step recovery option as a central component, not all evidence-based treatment follows this approach. As there are many people with former alcohol dependence who do not endorse the being ‘in recovery’ concept, there is strong argument for the need for treatment provision that does not insist on the adoption of this orientation in order to avoid unnecessarily excluding those in need of help [11].

There are a number of limitations with this research. First, the survey was retrospective, leading to the possibility of bias in recall of events that may have occurred decades in the past. Second, while a definition was provided to participants defining one drink as one unit of alcohol, it is possible that there were errors in self-reported alcohol consumption reported on the survey. Third, the definition of lifetime alcohol dependence reflected experience of symptoms throughout the participant’s life rather than experience of symptoms all within the same year. This can lead to an overly liberal definition of alcohol dependence that includes people with problems, that while still severe, may not reach the criteria of alcohol dependence as it would be measured in a clinical setting. Fourth, the section on treatment use employed a global treatment access question rather than asking each participant about use of different treatment services separately. This could lead to an underestimate of treatment use. Fifth, the project focussed on collecting a large representative sample of participants to estimate proportions of treated and untreated, and abstinent or moderate drinking remission. Another approach has been to recruit a smaller sample and to conduct in depth interviews to generate a more nuanced description of the patterns of remission among participants. This would also allow the measurement of some factors which, while important, were not included due to space limitations (e.g., use of other drugs by the participant before and after remission). Both approaches have their strengths and weaknesses and can be complementary to each other in order to develop a better understanding of remission. Finally, it is important to recognise that different methods of collecting representative samples each have their own limitations, with the current study employing an online panel. This approach generates a sample that matches the prevalence of certain demographic characteristics in the population but lacks a random selection element that is present in telephone and face-to-face survey methodology. This could mean that, even if the prevalence of some demographic characteristics are matched, other sample characteristics (such as past and current drinking) will not be present in the same proportions as those found in the general population. However, given that surveys which employed different participant recruitment methods have yielded a similar pattern of results as those observed in the present study, it may be reasonable to assume some confidence in the reliability of the current findings.

## Conclusions

Patterns of remission from alcohol dependence observed in the general population indicate a substantial number of people recovering without treatment and that many return to moderate drinking. It should be stressed that this broader, population health perspective does not contravene the value and need for treatment services, or that abstinence after remission is often the most valid clinical recommendation for those seeking help. However, this research is valuable because our understanding of the nature of addictions and their treatment can best be advanced from a foundation which incorporates an appreciation of the diversity of ways that people address their alcohol concerns.

### Electronic supplementary material

Below is the link to the electronic supplementary material.

Supplementary Material 1

## Data Availability

Available from the corresponding author on reasonable request.

## Abbreviations

AA: Alcoholics Anonymous
