# Supplementary material for: Patterns of remission from alcohol dependence in the United Kingdom: results from an online panel general population survey
Source: Subst Abuse Treat Prev Policy. 2024 Jan 4;19:3. doi: 10.1186/s13011-023-00588-1 (PMC10768276; doi:10.1186/s13011-023-00588-1)
Supplement: Supplementary file 1 — Supplementary Material 1 [file 13011_2023_588_MOESM1_ESM.pdf]

Manuscript Recovery Survey

Start of Block: Consent

Q3 Help us develop and evaluate online tools for people with alcohol concerns

Key Information and Consent:

Ethical Clearance Reference Number: HR-22/23-34937

You are invited to participate in this study that will help us understand people's experiences with alcohol. You do not need to be a current or past drinker to participate. Before you decide whether to you want to take part, it is important for you to understand why the research is being done and what your participation will involve.

Please take the time to read the following information carefully and discuss it with others if you wish.

What is the purpose of the study?
Take part in a study that will help us understand people's experiences with alcohol, how alcohol use might change over time, and how beliefs about alcohol use are related to consumption. The study will focus on how heavy drinkers have managed to reduce or quit drinking, and the development of online tools to support current heavy drinkers in reducing their alcohol consumption.

Why have I been invited to take part?
The study has been advertised on Prolific. You can choose to participate voluntarily if you are 18 years or older. You do not need to be a current or past drinker to participate.

What will happen if I take part?
If you agree to participate in the study, you will be asked to complete a survey containing a series of questions about people's drinking alcohol, some questions about your own current and past drinking experiences, as well as some demographic information about yourself (e.g., age, gender). Not everyone will see the same questions. The amount paid is UK Pounds Sterling **£**4.

Some participants will be asked if they are interested in taking part in another study at the completion of this survey. The additional study will ask for people's help to develop and evaluate online tools for people with alcohol concerns. We do not know if you will be asked to take part in this additional study. Those completing the additional study (which includes another survey after three months) will be paid an £2. If asked, you are completely free to refuse to take part in this additional study and your response will not impact in any way on your compensation for the

completing the current survey.

Duration and Privacy:
The survey will take you approximately up to 20-25 minutes to complete. Your responses will be kept completely anonymous, and we will not store any information that could be used to establish your identity. Any data that we collect will be stored securely on the KCL servers. Demographic information gathering is for research analysis and will not be identifiable. We take your privacy very seriously.

Do I have to take part?
Participation is completely voluntary. You should only take part if you want to and choosing not to take part will not disadvantage you in any way. It is possible that some people may experience discomfort in answering questions about their alcohol use. You are free to withdraw at any point during the completion of the study, without having to give a reason by simply not submitting the survey. Once you submit the survey, it will no longer be possible to withdraw from the study because the data will be anonymous.

What if I have further questions, or if something goes wrong?
If this study has harmed you in any way or if you wish to make a complaint about the conduct of the study you can contact KCL using the details below for further advice and information:

The Chair – Dr Karen Gillet
Email: rec@kcl.ac.uk

Contact Information:
If you have any questions, require more information about this study, or think you have been harmed by participating please contact the primary researcher using the following contact details:

John Cunningham, Addictions Sciences Building, 4 Windsor Walk, Denmark Hill, London SE5 8BB
Email: john.cunningham@kcl.ac.uk

Thank you!

Q4 By checking the box, you are confirming that you are 18 years or older, have read and understood the above information, and you agree to take part in the research. Your participation is voluntary and you are free to leave at any time by simply closing the web browser.

○ I agree to participate (1)

End of Block: Consent

Start of Block: AUDIT

Q377 First, please tell us your Prolific ID number?

____

Standard Drink Text Some questions in this questionnaire ask about “how many drinks”. For these questions, “one drink” equals one unit.

Audit1 How often did you have a drink containing alcohol during the past 12 months?

- ○ Never (0)
- ○ Monthly or less (1)
- ○ 2 to 4 times a month (2)
- ○ 2 to 3 times a week (3)
- ○ 4 to 5 times a week (4)
- ○ 6 or more times a week (5)

Display This Question:

If How often did you have a drink containing alcohol during the past 12 months? = Never

Drink_ever Have you EVER had a drink of any alcohol beverage?

- Yes (1)
- No (2)

Skip To: A6 If Have you EVER had a drink of any alcohol beverage? = Yes
Skip To: End of Block If Have you EVER had a drink of any alcohol beverage? = No

X→

Audit2 During the past 12 month, how many standard drinks containing alcohol did you have on a typical day when you were drinking?

- 1 to 2 (0)
- 3 to 4 (1)
- 5 to 6 (2)
- 7 to 9 (3)
- 10 to 12 (4)
- 13 to 15 (5)
- 16 or more (6)

X→

Audit3 During the past 12 months, how often did you have six or more standard drinks on one occasion?

- ○ Never (0)
- ○ Less than monthly (1)
- ○ Monthly (2)
- ○ Weekly (3)
- ○ Daily or almost daily (4)

Page Break

A6 Was there ever a time in your life when you drank six or more drinks on one occasion at least once a week, for at least a month or longer?

- Yes (1)
- No (2)

Display This Question:

If Have you EVER had a drink of any alcohol beverage? , Yes Is Not Displayed

A7 What is the largest number of drinks you can recall having on one occasion during the past 12 months?

____

Display This Question:

If Have you EVER had a drink of any alcohol beverage? , Yes Is Not Displayed

And If

During the past 12 month, how many standard drinks containing alcohol did you have on a typical d... != 1 to 2

Or During the past 12 months, how often did you have six or more standard drinks on one occasion? != Never

A8 How often did you have 12 or more drinks containing liquor of any kind on one occasion during the past 12 months?

- Never (0)
- More than once a day (1)
- About everyday (includes 6 times a week) (2)
- 4 to 5 times a week (3)
- 2 to 3 times a week (4)
- Once a week (5)
- 2 to 3 times a month (6)
- Once a month (7)
- Less than once a month (8)
- Don't know (99)

Page Break

Display This Question:

If Have you EVER had a drink of any alcohol beverage? , Yes Is Not Displayed

And If

During the past 12 month, how many standard drinks containing alcohol did you have on a typical d... != 1 to 2

Or During the past 12 months, how often did you have six or more standard drinks on one occasion? != Never

Audit4 How often during the last 12 months have you found that you were not able to stop drinking once you had started?

- Never (0)
- Less than monthly (1)
- Monthly (2)
- Weekly (3)
- Daily or almost daily (4)

Display This Question:

If Have you EVER had a drink of any alcohol beverage? , Yes Is Not Displayed

And If

During the past 12 month, how many standard drinks containing alcohol did you have on a typical d... != 1 to 2

Or During the past 12 months, how often did you have six or more standard drinks on one occasion? != Never

Audit5 How often during the last 12 months have you failed to do what was normally expected from you because of drinking?

- Never (0)
- Less than monthly (1)
- Monthly (2)
- Weekly (3)
- Daily or almost daily (4)

Page Break

Display This Question:

If Have you EVER had a drink of any alcohol beverage? , Yes Is Not Displayed

And If

During the past 12 month, how many standard drinks containing alcohol did you have on a typical d... != 1 to 2

Or During the past 12 months, how often did you have six or more standard drinks on one occasion? != Never

Audit6 How often during the last 12 months have you needed a first drink in the morning to get yourself going after a heavy drinking session?

- Never (0)
- Less than monthly (1)
- Monthly (2)
- Weekly (3)
- Daily or almost daily (4)

Display This Question:

If Have you EVER had a drink of any alcohol beverage? , Yes Is Not Displayed

And If

During the past 12 month, how many standard drinks containing alcohol did you have on a typical d... != 1 to 2

Or During the past 12 months, how often did you have six or more standard drinks on one occasion? != Never

Audit_7 How often during the last 12 months have you had a feeling of guilt or remorse after drinking?

- ○ Never (0)
- ○ Less than monthly (1)
- ○ Monthly (2)
- ○ Weekly (3)
- ○ Daily or almost daily (4)

Page Break

Display This Question:

If During the past 12 month, how many standard drinks containing alcohol did you have on a typical d... != 1 to 2

Or During the past 12 months, how often did you have six or more standard drinks on one occasion? != Never

And If

Have you EVER had a drink of any alcohol beverage? , Yes Is Not Displayed

Audit_8 How often during the last 12 months have you been unable to remember what happened the night before because you had been drinking?

- Never (0)
- Less than monthly (1)
- Monthly (2)
- Weekly (3)
- Daily or almost daily (4)

Audit_9 Have you or someone else ever been injured as a result of your drinking?

- No (0)
- Yes, but not in the last 12 months (1)
- Yes, during the last 12 months (2)

Audit_10 Has a relative or friend or a doctor or another health worker ever been concerned about your drinking or suggested you cut down?

- ○ No (0)
- ○ Yes, but not in the last 12 months (1)
- ○ Yes, during the last 12 months (2)

End of Block: AUDIT

Start of Block: Ask next set of questions to participants categorized as alcohol_cat = 2 or 3

Q29 For the next series of questions about your drinking, please think about the time in your life when you were drinking the heaviest.

PAudit_1 When your drinking was *at its heaviest*, how often did you have a drink containing alcohol?

- ○ Monthly or less (1)
- ○ 2 to 4 times a month (2)
- ○ 2 to 3 times a week (3)
- ○ 4 to 5 times a week (4)
- ○ 6 or more times a week (5)

PAudit_2 When your drinking was *at its heaviest*, how many standard drinks containing alcohol did you have on a typical day when you are drinking?

- 1 to 2 (0)
- 3 to 4 (1)
- 5 to 6 (2)
- 7 to 9 (3)
- 10 to 12 (4)
- 13 to 15 (5)
- 16 or more (6)

PAudit_3 When your drinking was *at its heaviest*, how often did you have six or more standard drinks on one occasion?

- Less than monthly (1)
- Monthly (2)
- Weekly (3)
- Daily or almost daily (4)

Page Break

PA7 When your drinking was *at its heaviest*, what was the *largest number* of drinks you can recall having on one occasion?

____

PA8 When your drinking was *at its heaviest*, how often did you have 12 OR MORE drinks containing liquor of any kind on ONE occasion?

- ○ Never (0)
- ○ More than once a day (1)
- ○ About everyday (includes SIX times a week) (2)
- ○ 4 to 5 times a week (3)
- ○ 2 to 3 times a week (4)
- ○ Once a week (5)
- ○ 2 to 3 times a month (6)
- ○ Once a month (7)
- ○ Less than once a month (8)
- ○ Don't know (99)

Page Break

PA9 How old were you when you started drinking at your heaviest?

____

PA10 How old were you when you stopped drinking this heavily?

____

PA11 At the time of your ***heaviest drinking***, did you *usually* drink alone or with other people?

- ○ Alone (1)
- ○ With others (2)
- ○ Both (3)
- ○ Don't know (99)

End of Block: Ask next set of questions to participants categorized as alcohol_cat = 2 or 3

Start of Block: Ask next set of questions to participants categorized as alcohol_cat = 3

FAA0A You have told us that you used to drink alcohol. Can you please tell us what lead you to stop drinking?

____

____

____

____

____

FAA0B In order to help us design more effective ways to help people with their drinking, we are interested in anything that you can tell us about what helped you to stop drinking. Can you tell us about any strategies you used to help you stop drinking?

____

____

____

____

____

FAA0C Some people try to quit drinking but are not successful. What helped you make this change more permanent?

____

____

____

____

____

Page Break

FAA1 Did you use to have a problem with alcohol but no longer do?

- Yes (1)
- No (2)
- Don't know (99)

FAA2 Do you consider yourself to be in recovery?

- Yes (1)
- No (2)
- Don't know (99)

FAA3 Did you ever consider yourself to be in recovery?

- Yes (1)
- No (2)
- Don't know (99)

Page Break

FAA5 Which of the following best describes how much of a problem your drinking was *at its heaviest?*

- Not a problem at all (1)
- Very minor problem (2)
- A minor problem (3)
- Major problem (4)
- Very major problem (5)
- Don't know (99)

FAA6 Before you stopped drinking, how many serious attempts to quit did you make? A serious quit attempt is one where you were really trying hard to quit and it lasted for at least 24 hours. Not drinking when sick is not a serious quit attempt.

____

FAA7 How old were you when you successfully quit drinking?

____

Page Break

FAA8 Did you quit all at once, or did you gradually cut down before you stopped altogether?

- All at once (1)
- Gradually cut down then stopped (2)
- I'm still drinking (3)
- Don't know (99)

Skip To: End of Block If Did you quit all at once, or did you gradually cut down before you stopped altogether? = I'm still drinking

FAA9 Did any of your family or friends encourage, help, or support you when you quit drinking?

- Yes (1)
- No (2)
- I'm still drinking (3)
- Don't know (99)

Skip To: End of Block If Did any of your family or friends encourage, help, or support you when you quit drinking? = I'm still drinking

FAA10 At the time you quit drinking, had any of your family or friends been pressuring you to quit?

- Yes (1)
- No (2)
- Don't know (99)

End of Block: Ask next set of questions to participants categorized as alcohol_cat = 3

Start of Block: Ask next set of questions to participants categorized as alcohol_cat = 2

Display This Question:

If Did you use to have a problem with alcohol but no longer do? , Yes Is Not Displayed

FAM0A You have told us that you used to drink more alcohol. In your own words, can you please tell us what lead you to reduce your drinking?

____

____

____

____

____

Display This Question:

If Did you use to have a problem with alcohol but no longer do? , Yes Is Not Displayed

FAM0B In order to help us design more effective ways to help people with their drinking, we are interested in anything you can tell us that helped you to reduce your drinking. Can you tell us about any strategies you used to help you reduce your drinking?

____

____

____

____

____

Display This Question:

If Did you use to have a problem with alcohol but no longer do? , Yes Is Not Displayed

FAM0C Some people try to reduce their drinking but are not successful. What helped you make this change more permanent?

____

____

Page Break

Display This Question:

If Did you use to have a problem with alcohol but no longer do? , Yes Is Not Displayed

FAM1 Did you use to have a problem with alcohol but no longer do?

- Yes (1)
- No (2)
- Don't know (99)

Display This Question:

If Did you use to have a problem with alcohol but no longer do? , Yes Is Not Displayed

FAM2 Do you consider yourself to be in recovery?

- Yes (1)
- No (2)
- Don't know (99)

Display This Question:

If Did you use to have a problem with alcohol but no longer do? , Yes Is Not Displayed

FAM3 Did you ever consider yourself to be in recovery?

- Yes (1)
- No (2)
- Don't know (99)

Page Break

Display This Question:

If Did you use to have a problem with alcohol but no longer do? , Yes Is Not Displayed

FAM5 Which of the following best describes how much of a problem your drinking was *at its heaviest?*

- ◯ Not a problem at all (1)
- ◯ Very minor problem (2)
- ◯ A minor problem (3)
- ◯ Major problem (4)
- ◯ Very major problem (5)
- ◯ Don't know (99)

Display This Question:

If Did you use to have a problem with alcohol but no longer do? , Yes Is Not Displayed

FAM6 Before you reduced your drinking to its current level, how many serious attempts did you make to change your drinking? A serious quit attempt is one where you were really trying hard to change your drinking and it lasted for at least 24 hours. Not drinking when sick is not a serious quit attempt.

____

Display This Question:

If Did you use to have a problem with alcohol but no longer do? , Yes Is Not Displayed

FAM7 How old were you when you successfully reduced your drinking to its current level?

____

Page Break

Display This Question:

If Did you use to have a problem with alcohol but no longer do? , Yes Is Not Displayed

Or Did you quit all at once, or did you gradually cut down before you stopped altogether? = I'm still drinking

FAM8 Did you reduce your drinking to its current level all at once or did you gradually cut down to your current level?

- All at once (1)
- Gradually cut down (2)
- I have not changed my level of drinking (3)
- Don't know (99)

Display This Question:

If Did you use to have a problem with alcohol but no longer do? , Yes Is Not Displayed

Or Did you quit all at once, or did you gradually cut down before you stopped altogether? = I'm still drinking

Or Did any of your family or friends encourage, help, or support you when you quit drinking? = I'm still drinking

FAM9 Did any of your family or friends encourage, help, or support you while you were reducing the amount you were drinking?

- Yes (1)
- No (2)
- I have not changed my level of drinking (3)
- Don't know (99)

Display This Question:

If Did you use to have a problem with alcohol but no longer do? , Yes Is Not Displayed

Or Did you quit all at once, or did you gradually cut down before you stopped altogether? = I'm still drinking

Or Did any of your family or friends encourage, help, or support you when you quit drinking? = I'm still drinking

FAM10 At the time you reduced your drinking, had any of your family or friends been pressuring you to change?

- ◯ Yes (1)
- ◯ No (2)
- ◯ Don't know (99)

End of Block: Ask next set of questions to participants categorized as alcohol_cat = 2

Start of Block: Ask next set of questions if A6=1 or categorized as alcohol_cat 4 or 5

Q48 Please tell us if the following has ever happened to you.

S1 Have you ever felt a very strong urge or desire to drink? By "strong urge" we mean "craving".

- ◯ Yes (1)
- ◯ No (2)
- ◯ Don't know (99)

Skip To: S2 If Have you ever felt a very strong urge or desire to drink? By "strong urge" we mean "craving". != Yes

S1a How old were you the first time you felt a very strong urge or desire to drink? If you are not sure, please take your best guess at your age when this first happened.

____

Display This Question:

If How often did you have a drink containing alcohol during the past 12 months? != Never

S1b When was the last time you felt a very strong urge or desire to drink?

- ◯ Less than one month ago (1)
- ◯ Between one and five months (2)
- ◯ Six months to one year (3)
- ◯ More than a year ago (4)
- ◯ Don't know (99)

Skip To: S2 If When was the last time you felt a very strong urge or desire to drink? != More than a year ago

S1c How old were you the last time you felt this way? That is, felt a very strong urge or desire to drink.

____

Page Break

S2 Have you ever started drinking even though you had decided not to?

◯ Yes (1)

◯ No (2)

◯ Don't know (99)

Skip To: S3 If Have you ever started drinking even though you had decided not to? != Yes

S2a How old were you the first time you started drinking even though you had decided not to? If you are not sure, please take your best guess at your age when this first happened.

____

Display This Question:

If How often did you have a drink containing alcohol during the past 12 months? != Never

S2b When was the last time you started drinking even though you had decided not to?

◯ Less than one month ago (1)

◯ Between one and five months (2)

◯ Six months to one year (3)

◯ More than a year ago (4)

◯ Don't know (99)

Skip To: S3 If When was the last time you started drinking even though you had decided not to? != More than a year ago

S2c How old were you the last time you did this? That is, started drinking even though you had decided not to.

____

Page Break

S3 Did you ever drink much more, or for much longer, than you had intended to?

- Yes (1)
- No (2)
- Don't know (99)

Skip To: S4 If Did you ever drink much more, or for much longer, than you had intended to? != Yes

S3a How old were you the first time you drank much more, or for much longer, than you had intended to?

____

Display This Question:

If How often did you have a drink containing alcohol during the past 12 months? != Never

S3b When was the last time you drank much more, or for much longer, than you had intended to?

- Less than one month ago (1)
- Between one and five months (2)
- Six months to one year (3)
- More than a year ago (4)
- Don't know (99)

Skip To: S4 If When was the last time you drank much more, or for much longer, than you had intended to? != More than a year ago

*

S3c How old were you the last time this happened? That is, drank much more, or for much longer, than you had intended to.

____

Page Break

S4 Have you ever tried to quit or cut down on your drinking but found you could not?

- Yes (1)
- No (2)
- Don't know (99)

Skip To: S5 If Have you ever tried to quit or cut down on your drinking but found you could not? != Yes

S4a How old were you the first time you tried to quit or cut down on your drinking but found you could not?

____

Display This Question:

If How often did you have a drink containing alcohol during the past 12 months? != Never

S4b When was the last time you tried to quit or cut down on your drinking but found you could not?

- Less than one month ago (1)
- Between one and five months (2)
- Six months to one year ago (3)
- More than a year ago (4)
- Don't know (99)

Skip To: S5 If When was the last time you tried to quit or cut down on your drinking but found you could not? != More than a year ago

*

S4c How old were you the last time you tried to do this? That is, to stop or cut down on your drinking but found you could not?

____

Page Break

S5 Have you ever felt sick or found yourself shaking when you cut down or quit drinking?

- Yes (1)
- No (2)
- Don't know (99)

Skip To: S6 If Have you ever felt sick or found yourself shaking when you cut down or quit drinking? != Yes

S5a How old were you the first time you felt sick or found yourself shaking when you cut down or quit drinking?

____

Display This Question:

If How often did you have a drink containing alcohol during the past 12 months? != Never

S5b When was the last time you felt sick or found yourself shaking when you cut down or quit drinking?

- Less than one month ago (1)
- Between one and five months (2)
- Six months to one year (3)
- More than a year ago (4)
- Don't know (99)

Skip To: S6 If When was the last time you felt sick or found yourself shaking when you cut down or quit drinking? != More than a year ago

*

S5c How old were you the last time this happened? That is, felt sick or found yourself shaking when you cut down or quit drinking.

____

Page Break

S6 Have you ever taken a drink (of alcohol) to get over any of the bad after effects of drinking?

◯ Yes (1)

◯ No (or have never had bad effects after drinking) (2)

◯ Don't know (99)

Skip To: S7 If Have you ever taken a drink (of alcohol) to get over any of the bad after effects of drinking? != Yes

S6a How old were you the first time you took a drink (of alcohol) to get over any of the bad after effects of drinking?

____

Display This Question:

If How often did you have a drink containing alcohol during the past 12 months? != Never

S6b When was the last time you took a drink (of alcohol) to get over any of the bad after effects of drinking?

◯ Less than one month ago (1)

◯ Between one and five months (2)

◯ Six months to one year (3)

◯ More than a year ago (4)

◯ Don't know (99)

Skip To: S7 If When was the last time you took a drink (of alcohol) to get over any of the bad after effects of... != More than a year ago

*

S6c How old were you the last time this happened? That is, took a drink (of alcohol) to get over any of the bad after effects of drinking.

____

Page Break

S7 Have you ever found that your usual number of drinks had much less effect on you than it once did?

- Yes (1)
- No (2)
- Don't know (99)

Skip To: S8 If Have you ever found that your usual number of drinks had much less effect on you than it once did? != Yes

S7a How old were you the first time you found that your usual number of drinks had much less effect on you than it once did?

____

Display This Question:

If How often did you have a drink containing alcohol during the past 12 months? != Never

S7b When was the last time you found that your usual number of drinks had much less effect on you than it once did?

- Less than one month ago (1)
- Between one and five months (2)
- Six months to one year (3)
- More than a year ago (4)
- Don't know (99)

Skip To: S8 If When was the last time you found that your usual number of drinks had much less effect on you tha... != More than a year ago

*

S7c How old were you the last time this happened? That is, you found that your usual number of drinks had much less effect on you than it once did.

____

Page Break

S8 Have you ever given up doing things you enjoy in favour of drinking?

- ◯ Yes (1)
- ◯ No (2)
- ◯ Don't know (99)

Skip To: S9 If Have you ever given up doing things you enjoy in favour of drinking? != Yes

S8a How old were you the first time you gave up doing things you enjoy in favour of drinking?

____

Display This Question:

If How often did you have a drink containing alcohol during the past 12 months? != Never

S8b When was the last time you gave up doing things you enjoy in favour of drinking?

- ◯ Less than one month ago (1)
- ◯ Between one and five months (2)
- ◯ Six months to one year (3)
- ◯ More than a year ago (4)
- ◯ Don't know (99)

Skip To: S9 If When was the last time you gave up doing things you enjoy in favour of drinking? != More than a year ago

S8c How old were you the last time this happened? That is, gave up doing things you enjoy in favour of drinking.

____

Page Break

S9 Have you ever spent a lot of your time on drinking, or getting over its effects, or doing things to get alcohol?

◯ Yes (1)

◯ No (2)

◯ Don't know (99)

Skip To: S10 If Have you ever spent a lot of your time on drinking, or getting over its effects, or doing things... != Yes

S9a How old were you the first time you spent a lot of your time on drinking, or getting over its effects, or doing things to get alcohol?

____

Display This Question:

If How often did you have a drink containing alcohol during the past 12 months? != Never

S9b When was the last time you spent a lot of your time on drinking, or getting over its effects, or doing things to get alcohol?

◯ Less than one month ago (1)

◯ Between one and five months (2)

◯ Six months to one year (3)

◯ More than a year ago (4)

◯ Don't know (99)

Skip To: S10 If When was the last time you spent a lot of your time on drinking, or getting over its effects, or... != More than a year ago

S9c How old were you the last time you spent a lot of your time on this? That is, drinking, or getting over its effects, or doing things to get alcohol.

____

Page Break

S10 Have you ever kept on drinking even though you had a health problem caused by or made worse by drinking?

- Yes (1)
- No (2)
- Don't know (99)

Skip To: S11 If Have you ever kept on drinking even though you had a health problem caused by or made worse by dr... != Yes

S10a How old were you the first time you kept on drinking even though you had a health problem caused by or made worse by drinking?

____

Display This Question:

If How often did you have a drink containing alcohol during the past 12 months? != Never

S10b When was the last time you kept on drinking even though you had a health problem caused by or made worse by drinking?

- Less than one month ago (1)
- Between one and five months (2)
- Six months to one year (3)
- More than a year ago (4)
- Don't know (99)

Skip To: S11 If When was the last time you kept on drinking even though you had a health problem caused by or mad... != More than a year ago

S10c How old were you the last time you did this? That is, kept on drinking even though you had a health problem caused by or made worse by drinking.

____

Page Break

S11 Have you ever kept on drinking even though it was making you depressed, uninterested in things, suspicious or distrustful?

- ○ Yes (1)
- ○ No (2)
- ○ Don't know (99)

Skip To: End of Block If Have you ever kept on drinking even though it was making you depressed, uninterested in things, s... != Yes

S11a How old were you the first time you kept on drinking even though it was making you depressed, uninterested in things, suspicious or distrustful?

____

Display This Question:

If How often did you have a drink containing alcohol during the past 12 months? != Never

S11b When was the last time you kept on drinking even though it was making you depressed, uninterested in things, suspicious or distrustful?

- ○ Less than one month ago (1)
- ○ Between one and five months (2)
- ○ Six months to one year (3)
- ○ More than a year ago (4)
- ○ Don't know (99)

Skip To: End of Block If When was the last time you kept on drinking even though it was making you depressed, uninterested... != More than a year ago

*

S11c How old were you the last time you kept on doing this? That is, drink even though it was making you depressed, uninterested in things, or suspicious or distrustful.

____

End of Block: Ask next set of questions if A6=1 or categorized as alcohol_cat 4 or 5

Start of Block: Treatment Questions

Tx1 This next question will ask about any help you might have received in relation to your drinking.

Have you every gone to Alcoholics Anonymous, or any other community agency or seen a physician, counselor, or any other professional for a reason that was related in any way to your drinking?

○ Yes (1)

○ No (2)

Skip To: Tx17 If This next question will ask about any help you might have received in relation to your drinking.... = No

Page Break

Tx2 Have you ever gone to Alcoholics Anonymous?

- ◯ Yes (1)
- ◯ No (2)
- ◯ Don't know (99)

Skip To: Tx3 If Have you ever gone to Alcoholics Anonymous? != Yes

Tx2a How old were you the first time you went to Alcoholics Anonymous?

____

Tx2b Some people attend Alcoholics Anonymous for a while, stop going and then start going to AA again at a later date. Roughly how many different PERIODS of time in your life have you gone to Alcoholics Anonymous for at least 3 meetings in a row?

____

Skip To: Tx2e If Condition: Some people attend Alcoholi... Is Equal to 0. Skip To: Was going to Alcoholics Anonymous ver....

Tx2c Was the longest period of time you went to Alcoholics Anonymous less than one month, between 1 and 6 months, between 7 and 11 months, between 1 and 5 years or for more than 5 years?

- ◯ Less than a month (1)
- ◯ 1-6 months (2)
- ◯ 7-11 months (3)
- ◯ 1-5 years (4)
- ◯ More than 5 years (5)
- ◯ Don't know (99)

Display This Question:

If If Some people attend Alcoholics Anonymous for a while, stop going and then start going to AA again... Text Response Is Not Equal to 1

And Was the longest period of time you went to Alcoholics Anonymous less than one month, between 1 an... != Less than a month

And Was the longest period of time you went to Alcoholics Anonymous less than one month, between 1 an... != 1-6 months

And Was the longest period of time you went to Alcoholics Anonymous less than one month, between 1 an... != 7-11 months

Tx2d How old were you the last time you went to Alcoholics Anonymous?

____

Tx2e Was going to Alcoholics Anonymous very helpful, somewhat helpful, or not helpful with your alcohol use?

- ◯ Very helpful (1)
- ◯ Somewhat helpful (2)
- ◯ Not helpful (3)
- ◯ Don't know (99)

Tx2f The last time you attended Alcoholics Anonymous, how acceptable did it feel for members to use medication as part of treatment for....

Alcohol or other drug concerns (Tx2f_1)	▼ Zero acceptance (1) ... Don't know - the topic never came up (5)
Mental health concerns (e.g., depression, anxiety or other mental health condition)? (Tx2f_2)	▼ Zero acceptance (1) ... Don't know - the topic never came up (5)
A physical health concern (e.g., hypertension, diabetes or other physical health condition)? (Tx2f_3)	▼ Zero acceptance (1) ... Don't know - the topic never came up (5)

Page Break

Tx3 Have you ever stayed overnight in a hospital for detoxification or related problems?

- Yes (1)
- No (2)
- Don't know (99)

Skip To: Tx4 If Have you ever stayed overnight in a hospital for detoxification or related problems? != Yes

Tx3a How old were you the first time you stayed overnight in a hospital for detoxification or related problems?

____

Tx3b Roughly how many TIMES would you say you stayed overnight in a hospital for detoxification or related problems?

____

Display This Question:

If If Roughly how many TIMES would you say you stayed overnight in a hospital for detoxification or rel... Text Response Is Not Equal to 1

Tx3d How old were you the last time you stayed overnight in a hospital for detoxification or related problems?

____

Tx3e Was staying overnight in a hospital for detoxification very helpful, somewhat helpful, or not helpful with your alcohol use?

- ○ Very helpful (1)
- ○ Somewhat helpful (2)
- ○ Not helpful (3)
- ○ Don't know (99)

Page Break

Tx4 Have you ever received treatment at a short-term inpatient alcohol treatment facility where you stay overnight and the program runs between 3 days to 3 months in length?

- ○ Yes (1)
- ○ No (2)
- ○ Don't know (99)

Skip To: Tx5 If Have you ever received treatment at a short-term inpatient alcohol treatment facility where you s... != Yes

Tx4a How old were you the first time you received alcohol treatment at an inpatient short-term alcohol treatment facility?

____

Tx4b How many different TIMES in your life have you received alcohol treatment at an inpatient short-term alcohol treatment facility?

____

Tx4c Did you ever stay for an entire program or did you leave before the program was completed?

- Yes: always stayed for entire program (1)
- Sometimes stayed for entire program, sometimes left before completed (2)
- No: always left before program completed (3)
- Not a program with a defined end point (4)
- Don't know (99)

Display This Question:

If If How many different TIMES in your life have you received alcohol treatment at an inpatient short-t... Text Response Is Not Equal to 1

Tx4d How old were you the last time you received alcohol treatment at an inpatient short-term alcohol treatment facility?

____

Tx4e Was receiving this treatment very helpful, somewhat helpful, or not helpful with your alcohol use?

- Very helpful (1)
- Somewhat helpful (2)
- Not helpful (3)
- Don't know (99)

Page Break

Tx5 Have you ever received alcohol treatment at a long-term residential program or therapeutic community? That is, in a program where most people stay more than 3 months?

- ○ Yes (1)
- ○ No (2)
- ○ Don't know (99)

Skip To: Tx6 If Have you ever received alcohol treatment at a long-term residential program or therapeutic commun... != Yes

Tx5a How old were you the first time you received alcohol treatment at a long-term residential program or therapeutic community?

____

Tx5b How many different times in your life have you received alcohol treatment at a long-term residential program or therapeutic community?

____

Tx5c Did you ever stay for an entire program or did you leave before the the program was completed?

- Yes: always stayed for entire program (1)
- Sometimes stayed for entire program, sometimes left before completed (2)
- No: always left before program completed (3)
- Not a program with a defined end point (4)
- Don't know (99)

Display This Question:

If If How many different times in your life have you received alcohol treatment at a long-term resident... Text Response Is Not Equal to 1

Tx5d How old were you the last time you received alcohol treatment at a long-term residential program or therapeutic community?

____

Tx5e Was receiving this treatment very helpful, somewhat helpful, or not helpful with your alcohol use?

- Very helpful (1)
- Somewhat helpful (2)
- Not helpful (3)
- Don't know (99)

Page Break

Tx6 Have you ever received treatment at an assessment or outpatient alcohol treatment facility where you attend during the day but do not stay overnight?

- Yes (1)
- No (2)
- Don't know (99)

Skip To: Tx7 If Have you ever received treatment at an assessment or outpatient alcohol treatment facility where... != Yes

Tx6a How old were you the first time you received treatment at an assessment or outpatient alcohol treatment facility?

____

Tx6b How many different times in your life have you received treatment at an assessment or outpatient alcohol treatment facility?

____

Tx6c Did you ever stay for an entire program or did you leave before the program was completed?

- ◯ Yes: always stayed for entire program (1)
- ◯ Sometimes stayed for entire program, sometimes left before completed (2)
- ◯ No: always left before program completed (3)
- ◯ Not a program with a defined end point (4)
- ◯ Don't know (99)

Display This Question:

If If How many different times in your life have you received treatment at an assessment or outpatient... Text Response Is Not Equal to 1

Tx6d How old were you the last time you received treatment at an assessment or outpatient alcohol treatment facility?

____

Tx6e Was receiving this treatment very helpful, somewhat helpful, or not helpful with your alcohol use?

- ◯ Very helpful (1)
- ◯ Somewhat helpful (2)
- ◯ Not helpful (3)
- ◯ Don't know (99)

Page Break

Tx7 Have you ever received alcohol treatment at a mental health centre or facility as an outpatient?

- Yes (1)
- No (2)
- Don't know (99)

Skip To: Tx8 If Have you ever received alcohol treatment at a mental health centre or facility as an outpatient? != Yes

Tx7a How old were you the first time you received alcohol treatment at a mental health centre or facility as an outpatient?

____

Tx7b How many different times in your life have you received alcohol treatment at a mental health centre or facility as an outpatient?

____

Tx7c Did you ever stay for an entire program or did you leave before the program was completed?

- ◯ Yes: always stayed for entire program (1)
- ◯ Sometimes stayed for entire program, sometimes left before completed (2)
- ◯ No: always left before program completed (3)
- ◯ Not a program with a defined end point (4)
- ◯ Don't know (99)

Display This Question:

If If How many different times in your life have you received alcohol treatment at a mental health cent... Text Response Is Not Equal to 1

Tx7d How old were you the last time you received alcohol treatment at a mental health centre or facility as an outpatient?

____

Tx7e Was receiving this treatment at a mental health centre very helpful, somewhat helpful, or not helpful with your alcohol use?

- ◯ Very helpful (1)
- ◯ Somewhat helpful (2)
- ◯ Not helpful (3)
- ◯ Don't know (99)

Page Break

Tx8 Have you ever received alcohol treatment at an employee assistance program?

- ○ Yes (1)
- ○ No (2)
- ○ Don't know (99)

Skip To: Tx9 If Have you ever received alcohol treatment at an employee assistance program? != Yes

---

Tx8a How old were you the first time you received alcohol treatment at an employee assistance program?

____

Tx8b How many different times in your life have you received alcohol treatment at an employee assistance program?

____

Tx8c Did you ever stay for an entire program or did you leave before the program was completed?

- Yes: always stayed for entire program (1)
- Sometimes stayed for entire program, sometimes left before completed (2)
- No: always left before program completed (3)
- Not a program with a defined end point (4)
- Don't know (99)

Display This Question:

If If How many different times in your life have you received alcohol treatment at an employee assistan... Text Response Is Not Equal to 1

Tx8d How old were you the last time you received alcohol treatment at an employee assistance program?

____

Tx8e Was receiving this treatment at an employee assistance program very helpful, somewhat helpful, or not helpful with your alcohol use?

- Very helpful (1)
- Somewhat helpful (2)
- Not helpful (3)
- Don't know (99)

Page Break

Tx9 Have you ever received alcohol treatment at a family or marital counseling service?

- Yes (1)
- No (2)
- Don't know (99)

Skip To: Tx10 If Have you ever received alcohol treatment at a family or marital counseling service? != Yes

Tx9a How old were you the first time you received alcohol treatment at a family or marital counseling service?

____

Tx9b How many different times in your life have you received alcohol treatment at a family or marital counseling service?

____

Tx9c Did you ever stay for an entire program or did you leave before the program was completed?

- ◯ Yes: always stayed for entire program (1)
- ◯ Sometimes stayed for entire program, sometimes left before completed (2)
- ◯ No: always left before program completed (3)
- ◯ Not a program with a defined end point (4)
- ◯ Don't know (99)

Display This Question:

If If How many different times in your life have you received alcohol treatment at a family or marital... Text Response Is Not Equal to 1

Tx9d How old were you the last time you received alcohol treatment at a family or marital counseling service?

____

Tx9e Was receiving this treatment very helpful, somewhat helpful, or not helpful with your alcohol use?

- ◯ Very helpful (1)
- ◯ Somewhat helpful (2)
- ◯ Not helpful (3)
- ◯ Don't know (99)

Page Break

Tx10 Have you ever received alcohol treatment at an emergency room?

◯ Yes (1)

◯ No (2)

◯ Don't know (99)

Skip To: Tx11 If Have you ever received alcohol treatment at an emergency room? != Yes

Tx10a How old were you the first time you received alcohol treatment at an emergency room?

____

Tx10b How many different times in your life have you received alcohol treatment at an emergency room?

____

Display This Question:

If If How many different times in your life have you received alcohol treatment at an emergency room? Text Response Is Not Equal to 1

Tx10d How old were you the last time you received alcohol treatment at an emergency room?

____

Tx10e Was receiving emergency room service very helpful, somewhat helpful, or not helpful with your alcohol use?

- Very helpful (1)
- Somewhat helpful (2)
- Not helpful (3)
- Don't know (99)

Page Break

Tx11 Have you ever received alcohol treatment by a physician, psychiatrist, psychologist, social worker or other professional at their private office as opposed to a larger alcohol treatment organisation?

- Yes (1)
- No (2)
- Don't know (99)

Skip To: Tx12 If Have you ever received alcohol treatment by a physician, psychiatrist, psychologist, social worke... != Yes

Tx11f What type of professional was it?

- Physician (1)
- Psychiatrist (2)
- Psychologist (3)
- Social worker (4)
- Other Professional (specify) (5) ________________
- Don't know (99)

Tx11a How old were you the FIRST time you received any alcohol treatment by a professional in a private office?

____

Tx11b How many different times in your life have you received alcohol treatment by a professional in a private office?

____

Tx11c Did you continue with the treatment as long as the professional wanted you to stay or did you stop before they indicated your treatment was completed?

- Stayed as long as professional wanted (1)
- Left earlier (2)
- Professional had no set end point (3)
- Sometimes continued and sometimes stopped (4)
- Don't know (99)

Display This Question:

If If How many different times in your life have you received alcohol treatment by a professional in a... Text Response Is Not Equal to 1

Tx11d How old were you the last time you received alcohol treatment by a professional in a private office?

____

Tx11e Was receiving this treatment very helpful, somewhat helpful, or not helpful with your alcohol use?

- ○ Very helpful (1)
- ○ Somewhat helpful (2)
- ○ Not helpful (3)
- ○ Don't know (99)

Page Break

Tx12 Have you ever received alcohol treatment in a jail or prison?

- Yes (1)
- No (2)
- Don't know (99)

Skip To: Tx13 If Have you ever received alcohol treatment in a jail or prison? != Yes

Tx12a How old were you the first time you received alcohol treatment in jail or prison?

____

Tx12b How many different times in your life have you received alcohol treatment in a jail or prison?

____

Tx12c Did you complete the entire program or did you stop going before the program was completed?

- Yes: always stayed for entire program (1)
- Sometimes stayed for entire program, sometimes left before completed (2)
- No: always left before program completed (3)
- Not a program with a defined end point (4)
- Don't know (99)

Display This Question:

If If How many different times in your life have you received alcohol treatment in a jail or prison? Text Response Is Not Equal to 1

Tx12d How old were you the last time you received alcohol treatment in a jail or prison?

____

Tx12e Was receiving treatment in a jail or prison very helpful, somewhat helpful, or not helpful with your alcohol use?

- Very helpful (1)
- Somewhat helpful (2)
- Not helpful (3)
- Don't know (99)

Page Break

Tx13 Have you ever received alcohol treatment from a minister, priest, rabbi, clergy or spiritual leader?

- ◯ Yes (1)
- ◯ No (2)
- ◯ Don't know (99)

Skip To: Tx14 If Have you ever received alcohol treatment from a minister, priest, rabbi, clergy or spiritual leader? != Yes

Tx13a How old were you the first time you received alcohol treatment from a minister, priest, rabbi, clergy or spiritual leader?

____

Tx13b How many different times in your life have you received alcohol treatment from a minister, priest, rabbi, clergy or spiritual leader?

____

Display This Question:

If If How many different times in your life have you received alcohol treatment from a minister, priest... Text Response Is Not Equal to 1

Tx13d How old were you the last time you received alcohol treatment from a minister, priest, rabbi, clergy or spiritual leader?

____

Tx13e Was receiving this assistance very helpful, somewhat helpful, or not helpful with your alcohol use?

- Very helpful (1)
- Somewhat helpful (2)
- Not helpful (3)
- Don't know (99)

Page Break

Tx14 Have you ever received alcohol treatment at a drink driving program?

- ◯ Yes (1)
- ◯ No (2)
- ◯ Don't know (99)

Skip To: Tx15 If Have you ever received alcohol treatment at a drink driving program? != Yes

Tx14a How old were you the first time you received alcohol treatment at a drink driving program?

____

Tx14b How many different times in your life have you received alcohol treatment at a drink driving program?

____

Tx14c Did you ever stay for an entire program or did you leave before the program was completed?

- ◯ Yes: always stayed for entire program (1)
- ◯ Sometimes stayed for entire program, sometimes left before completed (2)
- ◯ No: always left before program completed (3)
- ◯ Not a program with a defined end point (4)
- ◯ Don't know (99)

Display This Question:

If If How many different times in your life have you received alcohol treatment at a drink driving program? Text Response Is Not Equal to 1

Tx14d How old were you the last time you received alcohol treatment at a drinking driving program?

____

Tx14e Was receiving this assistance very helpful, somewhat helpful, or not helpful with your alcohol use?

- ◯ Very helpful (1)
- ◯ Somewhat helpful (2)
- ◯ Not helpful (3)
- ◯ Don't know (99)

Page Break

Tx15 Have you ever received any medicines for your alcohol use - e.g., acamprosate (Campral), disulfiram (Antabuse), nalmefene (Selincro)?

- ◯ Yes (1)
- ◯ No (2)
- ◯ Don't know (99)

Skip To: Tx16 If Have you ever received any medicines for your alcohol use - e.g., acamprosate (Campral), disulfir... != Yes

Tx15f What type of medicines did you receive for your alcohol use?

____

Page Break

Tx15a How old were you the first time you received these medicines for your alcohol use?

____

Tx15b How many different times in your life did you receive these medicines for your alcohol use?

____

Tx15c What was the longest period of time you took these medicines for your alcohol use?

- Less than a month (1)
- 1-6 months (2)
- 7-11 months (3)
- 1-5 years (4)
- More than 5 years (100)
- Don't know (99)

Display This Question:

If If How many different times in your life did you receive these medicines for your alcohol use? Text Response Is Not Equal to 1

Tx15d How old were you the last time you received these medicines for your alcohol use?

____

Tx15e Were taking these medicines very helpful, somewhat helpful, or not helpful with your alcohol use?

- ◯ Very helpful (1)
- ◯ Somewhat helpful (2)
- ◯ Not helpful (3)
- ◯ Don't know (99)

Page Break

Tx16 Have you ever received alcohol treatment at some other place? By “place” we mean service, treatment, etc., other than those we have already asked about.

- Yes (1)
- No (2)
- Don't know (99)

Skip To: TxEnd If Have you ever received alcohol treatment at some other place? By “place” we mean service, treatme... != Yes

Tx16f What type of place was it?

____

Tx16a How old were you the first time you received alcohol treatment at this other place?

____

Tx16b How many different times in your life did you receive alcohol treatment at this other place?

____

Display This Question:

If If How many different times in your life did you receive alcohol treatment at this other place? Text Response Is Not Equal to 1

Tx16d How old were you the last time you received alcohol treatment at this other place?

____

Tx16e Was receiving this assistance very helpful, somewhat helpful, or not helpful with your alcohol use?

- ○ Very helpful (1)
- ○ Somewhat helpful (2)
- ○ Not helpful (3)
- ○ Don't know (99)

Page Break

TxEnd Some services for helping drinkers provide more than evidence-based treatment for alcohol concerns. During the most recent time you received help for your drinking, were you offered:

	Yes (1)	No (0)	Don't know (99)
Employment support – help finding a steady job (TxEnd_1)	◯	◯	◯
Housing support – help finding a steady place to live (TxEnd_2)	◯	◯	◯
Mental health support – help dealing with a mental health concern (TxEnd_3)	◯	◯	◯
Physical healthcare support – help to improve access to physical healthcare (TxEnd_4)	◯	◯	◯

Display This Question:

If Some services for helping drinkers provide more than evidence-based treatment for alcohol concern... != Employment support – help finding a steady job [Yes]

TXEnd.1N Would employment support have been helpful for you?

◯ Yes (1)

◯ No (0)

◯ Don't know (99)

Display This Question:

If Some services for helping drinkers provide more than evidence-based treatment for alcohol concern... != Housing support – help finding a steady place to live [Yes]

TXEnd.2N Would housing support have been helpful for you?

- Yes (1)
- No (0)
- Don't know (99)

Display This Question:

If Some services for helping drinkers provide more than evidence-based treatment for alcohol concern... != Mental health support – help dealing with a mental health concern [Yes]

TXEnd.3N Would mental health support have been helpful for you?

- Yes (1)
- No (0)
- Don't know (99)

Display This Question:

If Some services for helping drinkers provide more than evidence-based treatment for alcohol concern... != Physical healthcare support – help to improve access to physical healthcare [Yes]

TXEnd.4N Would physical healthcare support have been helpful for you?

- Yes (1)
- No (0)
- Don't know (99)

Page Break

Tx17 Have you ever had contact with, or used any of the following, specifically for alcohol concerns?

	Yes, in the past year (1)	Yes, but not in the past year (2)	Never (3)
Consulted a community pharmacist for help with drinking (1)	○	○	○
Talked with someone at your GP surgery (e.g., doctor or nurse) for help with drinking (2)	○	○	○
I want to indicate that I have read this question by checking never . (3)	○	○	○
Phoned a helpline for help with drinking (e.g. DrinkLine) (4)	○	○	○
An alcohol self-help book or booklet (5)	○	○	○
Visited a website for help with drinking (6)	○	○	○
Used an alcohol application ('app') on a handheld computer (smartphone, tablet, PDA) (7)	○	○	○

Page Break

Display This Question:

If Have you ever gone to Alcoholics Anonymous? != Yes

Tx18. Did you ever attend an Alcoholics Anonymous meeting, even if you did not stay?

- ○ Yes (1)
- ○ No (2)
- ○ Don't know (3)

Skip To: End of Block If Did you ever attend an Alcoholics Anonymous meeting, even if you did not stay? != Yes

Display This Question:

If Have you ever gone to Alcoholics Anonymous? != Yes

Tx18a Why did you decide to attend?

- ○ I was concerned about my own alcohol consumption. (1)
- ○ Someone else was concerned about my alcohol consumption and persuaded me to attend. (2)
- ○ I was concerned about someone else's alcohol consumption. (3)
- ○ I was just curious, so I decided to go to a meeting. (4)

Display This Question:

If Have you ever gone to Alcoholics Anonymous? != Yes

*

Tx18b How many meetings did you attend?

____

Display This Question:

If Have you ever gone to Alcoholics Anonymous? != Yes

Tx18c Why did you stop going, or decide not to go back? (check all that apply)

- ▢ I dealt with my concerns and did not need the meetings any longer. (1)
- ▢ The AA meetings were not a good match for what I was looking for (2)
- ▢ I did not feel safe at the meeting (3)
- ▢ I only went because I was curious (4)
- ▢ Other (5) ____

Display This Question:

If Why did you stop going, or decide not to go back? (check all that apply) = The AA meetings were not a good match for what I was looking for

Tx18c1 Why was it not a good match for you? (Please check all that apply)

- ▢ I did not want to be abstinent (1)
- ▢ The 12-step approach was not a good match for me (2)
- ▢ I was pressured to stop taking medication I found helpful (3)
- ▢ Other (4) ____

End of Block: Treatment Questions

Start of Block: Demographics

R1 Please think about what it would take for someone with a serious alcohol problem to successfully recovery. How important do you think each of the following services are for someone to successfully recover from an alcohol problem?

	Essential for successful recovery (1)	An advantage (but not essential) (2)	Not necessary for successful recovery (3)	Don’t know (4)
Evidence-based treatment for alcohol concerns (1)	○	○	○	○
Employment support – help finding a steady job if they need it (2)	○	○	○	○
Housing support – help finding a steady place to live if they need it (3)	○	○	○	○
Mental health support – help dealing with a mental health concern if they need it (4)	○	○	○	○
The check the response 'not necessary' to show that you are paying attention (5)	○	○	○	○
Physical healthcare support – help to improve access to physical healthcare if the need it (6)	○	○	○	○

R2 When someone had a serious alcohol problem but they have now achieved and maintained sobriety do you believe that it is necessary for them to never drink alcohol again? To maintain

sobriety after a serious alcohol problem (choose the one answer that you think is the most accurate):

- ○ You can never drink alcohol again (1)
- ○ You need to have an extended period (at least a year) without drinking any alcohol but then you can drink at a moderate level (2)
- ○ You need to drink no alcohol almost all the time but it is okay to have one drink on rare special occasions (e.g., as a toast at a wedding) (3)
- ○ You can drink at a moderate level (4)

Q300 Do you think that it would make it harder for people to deal with their alcohol concerns if it was widely published that some people with serious alcohol problems:

	Yes (1)	No (2)	Don't know (3)
Can drink in moderate/social manner after they have dealt with their alcohol concerns? (1)	○	○	○
Can deal with their alcohol concerns without seeking treatment? (2)	○	○	○

End of Block: Demographics

Start of Block: Demographics

Page Break

Q3.1
First, please tell us a little about yourself.

How old are you?
(Tap inside the box below to bring up a keyboard and enter your age)

____

Q3.4 Gender?

- ○ Male (1)
- ○ Female (2)
- ○ Non-binary (3)
- ○ Other (*please specify below*): (7) ____

Age_Edu How old were you when you completed your continuous full-time education?

- 15 or under (1)
- 16 (2)
- 17 (3)
- 18 (4)
- 19 or over (5)
- Still in high school (6)
- Still in college or university (7)
- Other (please specify): (8) ____

Relat What is your current relationship status?

- Married / Common Law / Same Sex Partner (1)
- Single (2)
- Separated (3)
- Divorced (4)
- Widowed (5)

Employ What is your current employment status?

- Full-time / Self-employed (1)
- Part-time / Self-employed (2)
- Disabled (3)
- Not Employed (4)
- Student (5)
- Retired (6)
- Homemaker (7)

Fam_inc What is your gross family income (i.e., before taxes)?

- Less than £15,000 (1)
- Between £15,000 & £29,000 (2)
- Between £30,000 & £49,000 (3)
- Between £50,000 & £79,000 (4)
- More than £80,000 (5)
- Don't know (99)

Affirm Your answer to this question will not impact on our payment to your Prolific account.

	Strongy Disagree					Strongly Agree		
	1	2	3	4	4	5	6	7
I answered all questions truthfully								

(1)	

End of Block: Demographics

Start of Block: Services

Q331 Thank you for taking part in this survey. If you are concerned about your use, or would like more information, please go to the NHS.UK website. Additional useful resources can be found at the drinkaware.co.uk website.

Please check the next button to be taken back to the Prolific website and to be paid for completing this survey.

End of Block: Services
